# Efficacy differences of electroacupuncture with single acupoint or matching acupoints for chemotherapy-induced nausea and vomiting: study protocol for a randomized controlled trial

**DOI:** 10.1186/s13063-017-2186-y

**Published:** 2017-10-13

**Authors:** Bo Chen, Yang Guo, Xue Zhao, Li-li Gao, Bo Li, Tian-yi Zhao, Qi-wen Zhang, Jin-xing Zou, Ming-yue Li, Yong-ming Guo, Yi Guo, Xing-fan Pan

**Affiliations:** 1 Acu-moxibustion and Tuina Department of Tianjin University of Traditional Chinese Medicine, Tianjin, 300193 China; 2 Acupuncture Research Center of Tianjin University of Traditional Chinese Medicine, 88 Yuquan Road, Nankai District, Tianjin, 300193 China; 3 The First Affiliated Hospital of Tianjin University of Traditional Chinese Medicine, Tianjin, 300193 China; 4 Clinical Practice Teaching Department of Tianjin University of Traditional Chinese Medicine, Tianjin, 300193 China

**Keywords:** Chemotherapy-induced nausea and vomiting, Acupuncture, Matching acupoints, Randomized controlled trial

## Abstract

**Background:**

Previous studies have shown that acupuncture is beneficial for the alleviation of chemotherapy-induced nausea and vomiting. However, there is a lack of clinical evidence concerning the effects of acupoint-matching on chemotherapy-induced nausea and vomiting.

**Methods/design:**

This is a parallel randomized controlled trial to evaluate the occurrence of nausea and vomiting after chemotherapy (the incidence of nausea and vomiting, frequency, VAS score, RINVR rating) as the main outcome for cancer. Quality of life, anxiety and depression scores are the secondary outcomes. Quality of life, anxiety and depression scores are the secondary phase. Use of remedy drugs, routine blood examination, and blood biochemical tests are the safety evaluation. We also compare the different effects of ST36 (single acupoint), CV12 (single acupoint), and ST36-CV12 matching groups.

**Discussion:**

The results of this trial are expected to explore the effects of matching different acupoints and to offer biologic plausibility for the use of acupuncture in the treatment of chemotherapy-induced nausea and vomiting (CINV).

**Trial registration:**

This trial is registered with clinicaltrials.gov NCT02195921, The date of registration was 17 July 2014.

**Electronic supplementary material:**

The online version of this article (doi:10.1186/s13063-017-2186-y) contains supplementary material, which is available to authorized users.

## Background

### Introduction

Vomiting and nauseas are common side effects of chemotherapy [1]. The management of these side effects is challenging because antiemetic drugs have adverse effects such as constipation [2]. Acupuncture, a non-pharmacologic therapy, may be helpful for patients to manage the side effects of chemotherapy [3]. It has been shown that acupuncture may reduce the side effects of chemotherapy such as vomiting, nausea, hot flashes, and fatigue [4–6]. Acupuncture treatment has been recommended since 1997 by the National Institutes of Health (NIH) to help patients undergoing chemotherapy deal with severe side effects like vomiting and nausea [7]. Electroacupuncture has been recommended on the basis of level 1 evidence for the prevention of chemotherapy-induced nausea and vomiting (CINV) [8]. Such evidence has displayed the efficacy of acupuncture; however, how to match the acupoints remains a problem for clinical treatment.

**Fig. 1:**
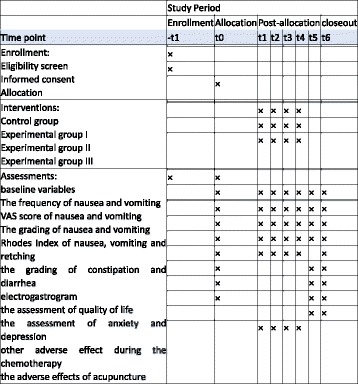
The schedule of enrollment, interventions, and assessments

Acupoints are the material basis for acupuncture effects [9], and the matching of acupoints is key to enhancing the effects. For single acupoint use, Zusanli (ST36) is one of the best acupoints for treatment of stomach disease, as it is beneficial for regulating the qi flow and harmonizing the stomach [10]. Zusanli (ST36) and Zhongwan (CV12) are considered as the most effective matching acupoints for the stomach qi on the basis of He-sea and Front-mu acupoint-matching (ST36 is the lower He-sea point of the stomach and CV12 is the Front-mu acupoint of the stomach) [11].

This randomized controlled trial evaluates the effects of acupuncture on CINV after chemotherapy to offer biologic plausibility for addressing vomiting and nausea. We will conduct this trial using ST36 single, CV12 single, and ST36-CV12 matching to investigate the quality of life scores, and depression/anxiety score as a secondary objective for patients undergoing chemotherapy as well as to explore the matching rules of acupoints.

## Methods

### Participants

This trial will be carried out at the Tianjin Medical University Cancer Institute and Hospital. All participants included in this trial are from the wards of integrated Chinese and western medicine, galactophore, lymphoma, and radiology.

### Study design

Random parallel control will be used as the clinical test design. Participants are randomly assigned to treatment groups: the ST36 single group (distal), CV12 single group (local), ST36-CV12 matching group (proximal-distal), and control group.

### Inclusion criteria

Inpatients from Tianjin Medical University Cancer Hospital who meet the following requirements will be eligible for enrollment: (1) diagnosed with cancer by imaging, cytology, pathology, and requiring chemotherapy; (2) Karnofsky (KPS) score ≥70 points; can take care of themselves; (3) no gender criteria, aged between 18 to 80; (4) inpatient or outpatient; (5) can be receiving single or multiple rounds of chemotherapy, but each patient should only be included once; (6) cancer patients undergoing chemotherapy combined with cisplatin (cisplatin ≥75 mg/m^2^) or anthracycline therapy (doxorubicin ≥40 mg/m^2^ or epirubicin ≥60 mg/m^2^); (7) expected lifespan longer than 6 months; (8) providing a signed hard copy of the agreement form.

### Exclusion criteria

Participants with any of the following conditions will be excluded: (1) radiotherapy and chemotherapy used at the same time; (2) nausea and vomiting due to cancers of the digestive system, such as gastric cancer; (3) chemotherapy patients with serious complications or severe liver/kidney function abnormalities [glutamic oxaloacetic transaminase (AST), glutamic-pyruvic transaminase (ALT), total bilirubin (TBIL) triple normal levels, blood urea nitrogen (BUN) and urine creatinine (Cr) double normal levels]; (4) fitted with pacemakers or other implanted medical electronic devices; (5) needle site with inflammation, scarring or trauma, or other serious systemic infection; (6) long-term use of opioids or metabolic imbalance (electrolyte imbalance) which cause vomiting; (7) a history of mental illness, language communication disorders; (8) postoperative patients with gastrointestinal obstruction and other mechanical risk factors; (9) patients with brain metastases or symptoms of increased intracranial pressure; (10) pregnancy or breast feeding.

### Interventions

A basic antiemetic plan (dexamethasone, ramosetron, or tropisetron) will be used in the control group. The remaining three groups will be given antiemetics and receive electroacupuncture at the ST36, CV12, and ST36-CV12 points. If vomiting cannot be controlled, a doctor will prescribe medication to relieve the symptoms according to the disease type.

### Control group

The control group will receive only a basic antiemetic regimen. This protocol will be delivered according to the American Society of Clinical Oncology clinical practice guidelines [12]. We chose 5-HT_3_ antagonists (ramosetron or tropisetron, beginning on day 1 of chemotherapy and taken continuously for 3–5 days) and dexamethasone.

### Experimental group

The acupoints are located according to the *WHO standard acupuncture point locations in the Western Pacific Region* [13]. Zusanli (ST36): below the knee, 3 *cun* below Dubi (ST35) on the line between Dubi (ST35) and Jiexi (ST41). Zhongwan (CV12): on the anterior midline of the abdomen, 4 cun above the umbilicus.

The needles (0.3 cun; Hua Tuo, Jiangsu, China) will be inserted and manipulated until De Qi (a sensation of soreness and tingling) is reported by the patient. Then the needle is connected to a Hua Tuo electroacupuncture therapy apparatus. Another refined electrode side will then be attached 1 cm beyond the acupoint. The frequency used is as in Shen et al. [14]; a bilateral 2-Hz current under 10 mA. The needles are left in place for 30 minutes. The treatment is delivered 30–60 minutes prior to commencement of chemotherapy. The therapy is delivered for 4 consecutive days.

### Outcomes

#### Primary outcome

The evaluation of nausea and vomiting is classified by the incidence of nausea and vomiting. In this trial, we use the Rhodes Index of Nausea, Vomiting and Retching [15] to score the incidence of nausea and vomiting. It could also be used to evaluate the duration, frequency, and severity of nausea and vomiting.

#### Secondary outcome

The Functional Assessment of Cancer Treatment (FACT) will be used to explore physical well-being (PWB), social well-being (SWB), emotional well-being (EWB), and functional well-being (FWB). The Hospital Anxiety and Depression Scale (HADS) will be used to identify anxiety and depression levels in the patients.

### Safety

Routine blood tests, liver/kidney function, constipation, diarrhea, and other antiemetic side effects, or adverse reactions will be assessed.

### Sample size calculation

Calculated using the mid-term results of our pilot study, the mean score for vomiting experience averaged over 5 days was 1.18 [weighted average standard deviation (SD) 0.53] in the CV12 group and 1.82 (weighted average SD 0.69) in the control group. At least 27 participants per arm would be required to detect this pair-wise difference between arms using a *t* test with a conservative Bonferroni-adjusted significance level of 0.05/6 = 0.0083 at a power of 90%. The power was for a two-tailed test of equal mean change scores at the 5% level of significance. Our study was planned to have high power to detect a two-unit difference in change scores. Thus, a total of 160 participants will be included in this trial with 40 patients in each group.

For the included studies, levels of attrition were noted. The impact of including studies with high levels of missing data in the overall assessment of treatment effect was explored by using sensitivity analysis. For all outcomes, analyses were carried out, as far as possible, on an intention-to-treat basis. The denominator for each outcome in each trial was the number randomly assigned minus any participants whose outcomes are known to be missing.

### Randomization and allocation concealment

This trial is randomized by a central randomization system. Eligible participants are randomly assigned to each group at a ratio of 1:1:1:1. Only the operational assistants log into http://www.tcmcec.net:8082/wcr/login.aspx and fill in the basic information of the subjects to apply a random number and distribution of constituencies. Stochastic systems are served by an independent third party, the Clinical Evaluation Center, China Academy of Traditional Chinese Medicine.

### Blinding

The participants, operational assistants, evaluators, and statisticians will be blinded to the treatment allocations, which will not be revealed until the end of the study. We have described for each included study the methods used to blind study participants and personnel from knowledge of which intervention a participant received. To prevent performance bias, studies are judged at low risk of bias if they were blinded or if we judged that the lack of blinding could not have affected the results. Evaluators and operational assistants will not work together at the same time. There will be no communication between patients. The evaluators will not be able to ask whether the patients receive the acupuncture or not or where the acupoints are. There will be no opportunities for the statisticians to become aware of the constituency distributions.

### Participant timeline

The enrollment will be carried out before the first day of chemotherapy on day 0. The electroacupuncture intervention will be given once daily from day 1 to day 4. All assessments will be scheduled from day 0 to day 5 (Fig. 1)

### Quality control

#### Clinical quality control

Level 1 examination (quality control) is conducted by the quality inspectors. They are required to develop quality checklists for all research data sources, data reporting, and adverse events. During the quality control process, they will need to take appropriate measures to assess quality problems and sign on the checklist. In Level 2 examinations (inspection), audit procedures and project sheets are in accordance with the progress of the completion of the test, the subjects in the group, and the relevant inspection project. The inspectors need to complete audit reports during the treatment. For the Level 3 examination (audit), case report form (CRF) management and assessment of the authenticity of the case will be carried out by the Clinical Evaluation Center, China Academy of Traditional Chinese Medicine.

### Document management and standardization of clinical treatment

To standardize clinical operations and deliver clinical quality assurance, we developed a series of documents and standardized clinical management operating specifications. Developing appropriate standard operating norms for various stages of clinical research is a way to ensure homogeneity between various researchers. It is helpful to use file management and develop a standard operating procedure (SOP) to ensure the feasibility, safety, and scientific integrity of clinical research.

### Data storage and confidentiality

The Clinical Evaluation Center, China Academy of Traditional Chinese Medicine provides a central stochastic system, database development, data entry and cleaning, data verification, statistical analysis, and other services as an independent third party. To reduce the risk of selection bias, the operator, evaluator, and statistician are separated.

### Statistical analysis

Statistical analysis will be conducted using SPSS (v.22; IBM Corp, Armonk, NY, USA). We will express normally distributed continuous outcomes using mean (SD) and skewed data with median (range). Dichotomous outcomes will be expressed as frequency or incidence. If the data are normally distributed, we will use ANOVA with Bonferroni-corrected pair-wise comparisons to compare the primary outcomes among the four treatment groups. If the data are not normally distributed, we will use a Kruskal-Wallis test to compare the four treatment groups and a Wilcoxon rank-sum test for the pair-wise comparison for the primary outcome. Intergroup differences in categorical data (Common Terminology Criteria for Adverse Events) will be assessed using the chi^2^ test or Fisher exact tests (two-tailed), as appropriate. Repeated measures analysis of variance (R-ANOVA) will be used to assess the frequency of vomiting, and the Visual Analogue Scale for nausea and nausea and vomiting and retching among the four study groups. Length of no days of nausea and vomiting will be calculated using Kaplan-Meier analysis and compared among the groups using the log-rank test. Subgroup analysis based on single or multiple chemotherapy sessions will be performed to compare the efficacy of basic antiemetic regimens plus electroacupuncture or an antiemetic regimen only on inducing a complete response. A *p* value <0.05 will be considered to be statistically significant.

## Discussion

The mechanism of CINV is complicated. What we know at present is that the 5-HT system and SP system may have an important role in CINV. 5-HT is mainly involved in the process of acute nausea and vomiting [15]. During chemotherapy, enterochromaffin cells (EC) release 5-HT receptors and vagal afferent nerves have been shown to be activated by 5-HT receptors. The signal is passed by the dorsal motor nucleus of the vagal nerve (vomiting center), which causes severe vomiting [16]. The SP system participates in the process, which prolongs the nausea and vomiting [17]. During long-term chemotherapy, cell apoptosis and inflammation induce EC to release P substances combined with NK-1 on the chemoreceptor trigger zone by the vagal afferent nerves. Then the signal reaches the stomach through the vagal efferent fibers, which delays nausea and vomiting [18]. It has been shown that the dopamine system plays an important role as well in the occurrence of CINV, but the specific mechanism is unclear [19].

Electroacupuncture, in particular, has been shown to inhibit SP and 5-HT release in EC. It may directly inhibit afferent vagal nerve excitability, or it may inhibit the solitary tract nucleus or vagal nerve dorsal nucleus as the acupuncture signals are conducted through the sensory nerves through the spinal cord upwards to the central level. Thus, CINV could be reduced. However, the choice of acupoints may also alter the efficacy of EA treatment. Clinical studies are still unable to provide guidance for the prevention of or treatment for CINV. Further, there is a lack of sufficient clinical evidence as to how acupoint-matching may improve CINV [20].

In terms of the risk of bias, derived from the partial absence of blinding of the interventionists, it is difficult to implement a fully blind method for the operators because acupuncture is a physical operation method, and the interventionists are required to be knowledgeable about acupuncture.

Nevertheless, the results of this trial are expected to explore the effects of the matching of different acupoints and offer biologic plausibility for the use of acupuncture in the treatment of CINV.

### Trial status

The trial is currently recruiting patients.

## Additional file

Additional file 1: SPIRIT 2013 Checklist. (DOCX 29 kb)

## Abbreviations

ALT: Glutamic-pyruvic transaminase
AST: Glutamic oxaloacetic transaminase
BUN: Blood urea nitrogen
CINV: Chemotherapy-induced nausea and vomiting
Cr: Urine creatinine
CRF: Case report form
CV12: The Zhongwan acupoint
FACT-G: Functional Assessment of Cancer Therapy - General
KPS: Karnofsky score
NIH: National Institutes of Health
ST36: The Zusanli acupoint
TBIL: Total bilirubin
