# Supplementary material for: Efficacy differences of electroacupuncture with single acupoint or matching acupoints for chemotherapy-induced nausea and vomiting: study protocol for a randomized controlled trial
Source: Trials. 2017 Oct 13;18:477. doi: 10.1186/s13063-017-2186-y (PMC5640953; doi:10.1186/s13063-017-2186-y)
Supplement: Additional file 1: — SPIRIT 2013 Checklist. (DOCX 29 kb) [file 13063_2017_2186_MOESM1_ESM.docx]

| Table 1. SPIRIT 2013 Checklist: Recommended Items to Address in a Clinical Trial Protocol and Related Documents* | | |
| --- | --- | --- |
| Section/Item | Item Number | Description |
|  |  |  |
| Administrativ e information | | |
| Title | 1 | Efficacy differences of electro-acupuncture with single acupoint or matching acupoints for chemotherapy-induced nausea and vomiting: Study protocol for a randomized controlled trial |
| Trial registration | 2a | clinicaltrials.gov NCT02195921 |
|  | 2b | https://register.clinicaltrials.gov/prs/app/action/SelectProtocol?sid=S0004ZHK&selectaction=Edit&uid=U0002CV6&ts=2&cx=19hfpo |
| Protocol version | 3 | Version1.0-20160801 |
| Funding | 4 | This work was supported in part by National Basic Research Program of China under Grant No. 2014CB543201. |
| Roles and responsibilities | 5 | Bo Chen＃1 2  Email: tjutcmchenbo@163.com  Yang Guo＃3  Email: guoguo_guoyang@hotmail.com  Xue Zhao＃1 2  Email: dreamj0216@126.com  Li-li Gao1 2  Email: 465208311@qq.com  Bo Li1 2  Email: bb2065402@163.com  Tian-yi Zhao1 2  Email: zhaotianyi89@126.com  Qi-wen Zhang1 2  Email: 626938768@qq.com  Jin-xing Zou1 2  Email: 1024749796@qq.com  Ming-yue Li4  Email: limingyue7@163.com  Yong-ming Guo1 2  Email: Guoymxr@163.com  Yi Guo*1 2  Email: guoyi_168@163.com  Xing-fan Pan*1 2  Email: panxingfang@163.com  1Acu-moxibustion and Tuina Department of Tianjin University of Traditional Chinese Medicine, Tianjin 300193, China.  2.Acupuncture Research Center of Tianjin University of Traditional Chinese Medicine, Tianjin 300193, China.  3. The First Affiliated Hospital of Tianjin University of Traditional Chinese Medicine, Tianjin 300193, China.  4.Clinical Practice Teaching Department of Tianjin University of Traditional Chinese Medicine, Tianjin 300193, China.  * Corresponding author. Acupuncture Research Center of Tianjin University of Traditional Chinese Medicine, 88 Yuquan Road, Nankai District, Tianjin, China,300193.  BC, YG and XZ contributed equally to this work. BC, XZ and XFP conceived this protocol. BC, YG and MYL composed this trial. LLG, BL and TYZ majorly participated in enrollment and clinical treatment. QWZ and JXZ are the clinical evaluator. YMG, 8ZLC is the clinical data monitors. YG is the project director and response for the top-level design. Correspondence should be addressed to YG and XFP. All authors read and approved the final manuscript. |
| Introduction |  |  |
| Background and rationale | 6 | Previous studies have shown that acupuncture is beneficial for the alleviation of chemotherapy-induced nausea and vomiting. However, there is a lack of clinical evidence concerning the effects of acupoint-matching on chemotherapy-induced nausea and vomiting. |
| Objectives | 7 | To evaluate the efficacy differences of electro-acupuncture with single acupoint or matching acupoints for chemotherapy-induced nausea and vomiting |
| Trial design | 8 | Random parallel control will be used as the clinical test design. Participants are randomly assigned to treatment groups: ST36 single group (distal), CV12 single group (local), ST36-CV12 matching group (proximal-distal) and control group. |
| Methods |  |  |
| Participants, interventions,and outcomes | | |
| Study setting | 9 | Inpatients from Tianjin Medcial University Cancer Hospital |
| Eligibility criteria | 10 | Inclusion Criteria :(1) diagnosed with cancer by imaging, cytology, pathology and requiring chemotherapy; (2) Karnofsky score ≥70 points; can take care of themselves. (KPS Scale see Appendix 2); (3) no gender criteria, aged between 18 to 80; (4) inpatient or outpatient; (5) can be receiving single or multiple rounds of chemotherapy, but each patient should only be included once. (6) cancer patients undergoing chemotherapy combined with cisplatin (cisplatin ≥75mg / m2) or anthracycline therapy (doxorubicin ≥40 mg / m2 or epirubicin ≥60 mg / m2); (7) expected lifespan longer than 6 months; (8) providing a signed hard-copy of the agreement form.  Exclusion Criteria: (1) radiotherapy and chemotherapy used at the same time; (2) have nausea and vomiting due to cancers of the digestive system, such as gastric cancer; (3) chemotherapy patients with serious complications or severe liver/kidney function abnormalities (AST, ALT, TBIL triple than normal, BUN and Cr double than normal); (4) fitted with pacemakers or other implanted medical electronic devices; (5) needling site with inflammation, scarring or trauma, or other serious systemic infection; (6) long-term use of opioids or metabolic imbalance (electrolyte imbalance) which cause vomiting; (7) a history of mental illness, language communication disorders; (8) postoperative patients with gastrointestinal obstruction and other mechanical risk factors; (9) patients with brain metastases or symptoms of increased intracranial pressure; (10) pregnancy or breast feeding. |
| Interventions | 11 | Interventions  A basic antiemetic plan (Dexamethasone, ramosetron, or tropisetron) will be used in the control group. The remaining three groups will be given antiemetics and receive electro-acupuncture at the ST36, CV12, ST36-CV12 points. If vomiting cannot be controlled, a doctor will prescribe medication to relieve the symptoms according to the disease type.  Control Group  The control group will receive only a basic antiemetic regimen. This protocol will be delivered according to the American Society of Clinical Oncology clinical practice guidelines. [12] We chose 5-HT3 antagonists (ramosetron or tropisetron, beginning on day 1 of chemotherapy and taken continuously for 3-5 days) and dexamethasone. Experimental Group  The acupoints are located according to the WHO standard acupuncture point locations in the Western Pacific Region. [13] Zusanli (ST36): below the knee, 3 cun below Dubi (ST35) on the line between Dubi (ST35) and Jiexi (ST41). Zhongwan (CV12): on the anterior midline of the abdomen, 4 cun above the umbilicus.  The needles (0.3 cun; Hua Tuo, Jiangsu, China) will be inserted and manipulated until De Qi (a sensation of soreness and tingling) is reported by the patient. Then the needle is connected to a Hua Tuo electro-acupuncture therapy apparatus. Another refined electrode side will then be attached 1 cm beyond the acupoint. The frequency used is as in Shen J, et al. [14]; a bilateral 2-Hz current under 10mA. The needles are left in place for 30 minutes. The treatment is delivered 30-60 minutes prior to commencement of chemotherapy. The therapy is delivered four consecutive days. |
| Outcomes | 12 | Primary Outcome  The evaluation of nausea and vomiting is classified by the incidence of nausea and vomiting. In this trial, we use the Rhodes Index of Nausea, Vomiting and Retching [15] to score the incidence of nausea and vomiting. It could also be used to evaluate the duration, frequency and severity of nausea and vomiting.  Secondary Outcome  Functional Assessment of Cancer Treatment (FACT) will be used to explore PWB, SWB, EWB, FWB. Hospital Anxiety and Depression Scale (HADS) will be used to identify anxiety and depression levels in the patients.  Safety |
| Participant timeline | 13 | The enrollment will be carried out before the first day of chemotherapy on day zero. The electroacupuncture intervention will be given once daily from day one to day four. All assessments will be scheduled from day zero to day five. An additional movie file shows this in more detail [see Additional file 1]. |
| Sample size | 14 | Calculated using the mid-term results of our pilot study, the mean score for vomiting experience averaged over 5 days was 1.18 [weighted average standard deviation (SD) 0.53] in the CV12 group and 1.82 (weighted average SD 0.69) in the control group. At least 27 participants per arm would be required to detect this pair-wise difference between arms using a t-test with a conservative Bonferroni adjusted significance level of 0.05/6 = 0.0083 at a power of 90%. The power was for a two-tailed test of equal mean change scores at the 5% level of significance. Our study was planned to have high power to detect a 2-unit difference in change scores. Thus, a total of 160 participants will be included in this trial with 40 patients in each group.  For included studies, levels of attrition were noted. The impact of including studies with high levels of missing data in the overall assessment of treatment effect was explored by using sensitivity analysis. For all outcomes, analyses were carried out, as far as possible, on an intention-to-treat basis. The denominator for each outcome in each trial was the number randomly assigned minus any participants whose outcomes are known to be missing. |
| Recruitment | 15 | In this study, our patients will spend 4-5 days in hospital for the first course because they must receive the basic treatment for cancer. These 4-5 days will ensure us to arrange the trial for them. We choose the follow-up period in their chemotherapy course because in that period they have good compliance. We allow a flexible treatment regimen that accommodates individual differences in efficacy and side effects in order to reduce the dropout rate because of a lack of efficacy or tolerability. And our follow-up period will be shorten just for 4 weeks. |
| Assignment of interventions (for controlled trials) | | |
| Allocation |  |  |
| Sequence generation  Allocation concealment mechanism  Implementation | 16 | This trial is randomized by a central randomization system. Eligible participants are randomly assigned to each group at a ratio of 1:1:1:1. Only the operational assistants log into http://www.tcmcec.net/crivrs/ and fill in the basic information of the subjects to apply random number and distribution of constituencies. Stochastic systems are served by an independent third party, the Clinical Evaluation Center, China Academy of Traditional Chinese Medicine. |
| Blinding (masking) | 17 | The participants, operational assistants, evaluators, and statisticians will be blinded to the treatment allocations, which will not be revealed until the end of the study. We have described for each included study the methods used to blind study participants and personnel from knowledge of which intervention a participant received. To prevent performance bias, studies are judged at low risk of bias if they were blinded or if we judged that the lack of blinding could not have affected the results. Evaluators and operational assistants will not work together at the same time. There will be no communication between patients. The evaluators will not be able to ask whether the patients receive the acupuncture or not or where the acupoints are. There will be no opportunities for the statisticians to become aware of the constituency distributions. |
| Data collection, management, and analysis | | |
| Data collection methods | 18a | Plans for assessment and collection of outcome, baseline, and other trial data, including any related processes to promote data quality (e.g., duplicate measurements, training of assessors) and a description of study instruments (e.g., questionnaires, laboratory tests) along with their reliability and validity, if known. Reference to where data collection forms can be found, if not in the protocol. |
|  | 18b | Plans to promote participant retention and complete follow-up, including list of any outcome data to be collected for participants who discontinue or deviate from intervention protocols |
| Data management | 19 | The Clinical Evaluation Center, China Academy of Traditional Chinese Medicine, provides a central stochastic system, database development, data entry and cleaning, data verification, statistical analysis and other services as an independent third party. To reduce the risk of selection bias, the operator, evaluator and statistician are separated. |
| Statistical methods | 20 | Statistical analysis will be conducted using SPSS (v.22). We will express normally distributed continuous outcomes using mean (SD) and skewed data with median (range). Dichotomous outcomes will be expressed as frequency or incidence. If the data are normally distributed, we will use ANOVA with Bonferroni corrected pair-wise comparisons to compare the primary outcomes among the 4 treatment groups. If the data are not normally distributed, we will use a Kruskal-Wallis test to compare the 4 treatment groups and a Wilcoxon rank-sum test for the pair-wise comparison for the primary outcome. Intergroup differences in categorical data (Common Terminology Criteria for Adverse Events) will be assessed using the chi2 test or Fisher exact tests (2-tailed), as appropriate. Repeated measures analysis of variance (R-ANOVA) will be used to assess the frequency of vomiting, Visual Analogue Scale nausea and of nausea and vomiting and retching among the 4 study groups. Length of no days of nausea and vomiting will be calculated using Kaplan-Meier analysis and compared among the groups using the log-rank test. Subgroup analysis based on single or multiple chemotherapy sessions will be performed to compare the efficacy of basic antiemetic regimens plus electro-acupuncture or an antiemetic regimen only on inducing a complete response. A p value <0.05 will be considered to be statistically significant. |
| Monitoring Data monitoring | 21 | Level 1 examination (quality control) is conducted by the quality inspectors. They are required to develop quality checklists for all research data sources, data reporting, and adverse events. During the quality control process, they will need to take appropriate measures to assess quality problems and sign on the checklist. In Level 2 examinations (inspection), audit procedures and project sheets are in accordance with the progress of the completion of the test, the subjects in the group and the relevant inspection project. The inspectors need to complete audit reports during the treatment. For the Level 3 examination (audit), CRF management and assessing the authenticity of the case will be carried out by the Clinical Evaluation Center, China Academy of Traditional Chinese Medicine.  To standardize clinical operations and deliver clinical quality assurance, we developed a series of documents and standardized clinical management operating specifications. Developing appropriate standard operating norms for various stages of clinical research is a way to ensure homogeneity between various researchers. It is helpful to use file management and development of a standard operating procedure (SOP) to ensure the feasibility, safety, and scientific integrity of clinical research. |
| Harms | 22 | Blood routine, liver/kidney function, constipation, diarrhea and other antiemetic side effects, or adverse reactions will collect, assess, report. |
| Auditing | 23 | Clinical Evaluation Center, China Academy of Traditional Chinese Medicine audits trial conduct independently one time a month. |
| Ethics and dissemination |  |  |
| Research ethics approval | 24 | This trial has been approved by the local institutional ethics committee (Tianjin University of TCM, Tianjin, China). The approval number is TJUTCM-EC20140005. |
| Protocol amendments | 25 | no |
| Consent or assent | 26a | Operational assistants will obtain informed consent |
| Confidentiality | 27 | personal information will be kept secret before, during, and after the trial. Their names will be stead of numbers. |
| Declaration of interests | 28 | no competing interests |
| Access to data | 29 | statistician will have access to the final trial data set. |
| Ancillary and post-trial care | 30 | compensation to those who suffer harm from trial participation |
| Dissemination policy | 31 | investigators and sponsor to communicate trial results to participants, health care professionals, the public, and other relevant groups via publication. |
| Appendices Informed consent materials | 32 | Model consent form and other related documentation given to participants and authorized surrogates |
| Biological specimens | 33 | no |
